# Insights into the Transport Cycle of LAT1 and Interaction with the Inhibitor JPH203

**DOI:** 10.3390/ijms24044042

**Published:** 2023-02-17

**Authors:** Chiara Brunocilla, Lara Console, Filomena Rovella, Cesare Indiveri

**Affiliations:** 1 Department DiBEST (Biologia, Ecologia, Scienze Della Terra) Unit of Biochemistry and Molecular Biotechnology, University of Calabria, Via Bucci 4C, 87036 Arcavacata di Rende, Italy; 2 CNR Institute of Biomembranes, Bioenergetics and Molecular Biotechnologies (IBIOM), Via Amendola 122/O, 70126 Bari, Italy

**Keywords:** conformational changes, in silico analysis, SLC7A5, transport cycle, transport proteins

## Abstract

The large Amino Acid Transporter 1 (LAT1) is an interesting target in drug discovery since this transporter is overexpressed in several human cancers. Furthermore, due to its location in the blood-brain barrier (BBB), LAT1 is interesting for delivering pro-drugs to the brain. In this work, we focused on defining the transport cycle of LAT1 using an in silico approach. So far, studies of the interaction of LAT1 with substrates and inhibitors have not considered that the transporter must undergo at least four different conformations to complete the transport cycle. We built outward-open and inward-occluded conformations of LAT1 using an optimized homology modelling procedure. We used these 3D models and the cryo-EM structures in outward-occluded and inward-open conformations to define the substrate/protein interaction during the transport cycle. We found that the binding scores for the substrate depend on the conformation, with the occluded states as the crucial steps affecting the substrate affinity. Finally, we analyzed the interaction of JPH203, a high-affinity inhibitor of LAT1. The results indicate that conformational states must be considered for in silico analyses and early-stage drug discovery. The two built models, together with the available cryo-EM 3D structures, provide important information on the LAT1 transport cycle, which could be used to speed up the identification of potential inhibitors through in silico screening.

## 1. Introduction

The human membrane amino acids transporter LAT1 (SLC7A5) is the fifth member of the SLC7 protein family that belongs to the large Amino acid-Polyamine-organo Cation (APC) superfamily. The proteins of the SLC7A5 family are classified into two subfamilies, the Cationic Amino acids Transporters (CATs) and the Heterodimeric Amino acids Transporters (HATs) [1]. The heterodimeric amino acid transporters are characterized by 12 trans-membrane domains, whereas the cationic amino acid transporters share 14 transmembrane domains. The peculiarity of the heterodimeric transporters is that of being constituted by a non-glycosylated subunit (LATs) and a glycosylated subunit, belonging to the SLC3 family containing a single transmembrane domain and a large extracellular domain. LAT1 belongs to the HAT subfamily and forms a heterodimer with the glycoprotein SLC3A2, also known as CD98. This heterodimer is characterized by a covalent bond between two conserved cysteines, C164 of the light subunit LAT1 and C109 of the heavy subunit CD98 [1,2,3,4,5]. CD98 is involved in the membrane trafficking [6,7] of LAT1, which is the sole subunit responsible for the transport function. It catalyzes an electroneutral antiport of amino acids with specificity for essential amino acids [1,2,3,4,5] and cysteine [8,9,10]. In vivo, histidine, one of the main substrates of this transporter, is most likely exported from cells that permit other amino acids to be absorbed [8,9,11]. Leucine is also a high-affinity substrate of LAT1; it is important in the amino acids sensing process involving mTORC1 [12,13].

The regulatory aspects of LAT1 biology are still poorly understood. Notable stimulation of transport activity was found in the presence of cholesterol at physiological concentrations. A synergistic effect by ATP was also observed only in the presence of cholesterol, with an increase in the substrate affinity only on the internal site [14]. LAT1 is mainly expressed in the blood-brain barrier (BBB) and placenta, where it regulates the homeostasis of essential amino acids [1,9]. Accordingly, impairment of LAT1 function due to gene defects causes alteration of amino acid homeostasis in the brain, leading to autism spectrum disorder in humans [11]. Similarly, knocking out the BBB LAT1 expression in mice led to a pathology mimicking the autism spectrum disorder [11]. Very importantly, LAT1 is overexpressed in most human cancers, even originating from tissues that normally do not express the transporter [9,15,16]. In this sense, LAT1 is an interesting target in human pharmacology. Indeed, many molecules acting as inhibitors of this transporter have been identified [17,18] to chemically knock out the transporter, which is specifically expressed in cancer cells. One of the identified compounds, namely JPH203, has reached clinical trials [19]. Since this molecule is a tyrosine derivative, it inhibits LAT1 in a competitive way with the transporter substrate. Moreover, LAT1 is a very good candidate for the pro-drug approach, in which a drug molecule is linked to the physiological substrate of the transporter for facilitating membrane crossing. This approach is particularly interesting since LAT1 is physiologically expressed at the BBB, thus giving a further possibility to deliver a drug into the brain [20]. Therefore, LAT1 is an important target for research in drug discovery. Many efforts are in the course for identifying additional inhibitors with higher affinity and lower side effects with respect to those available. For this purpose, in silico methodologies are mandatory for the early-stage screening of chemical libraries. However, LAT1, as most membrane transporters, must undergo at least four different structural conformations for transport to occur: one facing the extracellular side (outward open), allowing the substrate to bind to the transporter; a closed conformation still facing the extracellular side (outward occluded); a closed conformation facing towards the intracellular side (inward occluded); and, finally, a conformation open towards the intracellular side (inward open) allowing the substrate to be released into the cytosol [21,22]. The knowledge of these steps is mandatory not only for understanding the mechanism of the transport cycle but also for predicting and defining in detail the interaction with substrates and other kinds of molecules such as potential new drugs. We perform an in silico analysis for defining the major steps of the transport cycle of LAT1 and the interactions with the substrates. The obtained results correlate well with previously reported in vitro data.

## 2. Results

**Figure 1:**
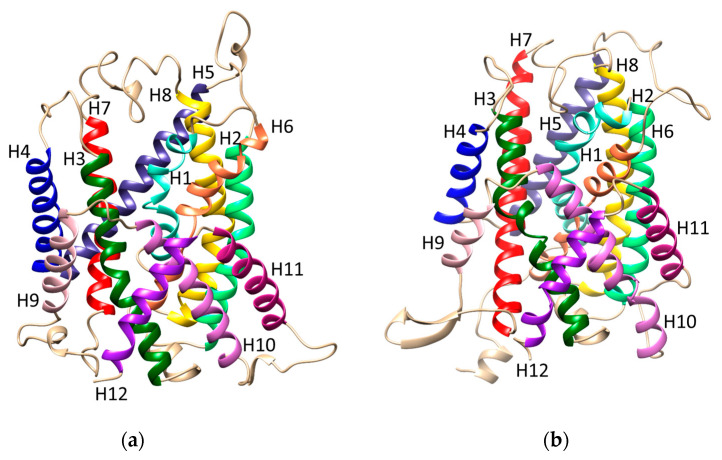
Ribbon representation of LAT1 homology model structures, lateral view of (**a**) the outward-open and (**b**) the inward-occluded conformations, after optimization with Molecular Dynamics. Transmembrane segments are indicated by (H). The extracellular side is the upper part.

Three-dimensional structures of LAT1 in the conformations outward occluded and inward open have been recently obtained using Cryo-EM and are available in the Protein Data Bank. We chose the structure with the PDB-ID 7DSK, JX-075-bound holo-conformation, and PDB-ID 6IRS apo-conformation for their higher resolution compared to other available structures [23,24]. However, the outward-open and inward-occluded conformations are essential steps of the transport cycle, as described for other membrane transporters [21,22,25]. Therefore, these two conformations have been predicted using homology modelling and optimized as described in the materials and methods to study the entire transport cycle. For suitable model structures, more than one template was chosen for each of the two conformations, and then molecular dynamics was performed to optimize the structures (see Materials and Methods Section 4.1). Figure 1 shows the outward-open (a) and the inward-occluded (b) conformation of LAT1 obtained using homology modeling. 

These two conformations represent the initial and one of the intermediate steps of the transport cycle (starting from outside the cell). Indeed, extensive conformational changes are evident in Figure 1a,b. The structural models show positions and/or orientation changes of at least half of the transmembrane α-helices (H), such as H1, H2, H4, H6, H9, and H11. The extensive moving represents an example of how the transporter undergoes the necessary conformational changes to perform the various steps of the transport cycle, as described below. 

### 2.1. Binding Site Characterization

**Figure 2:**
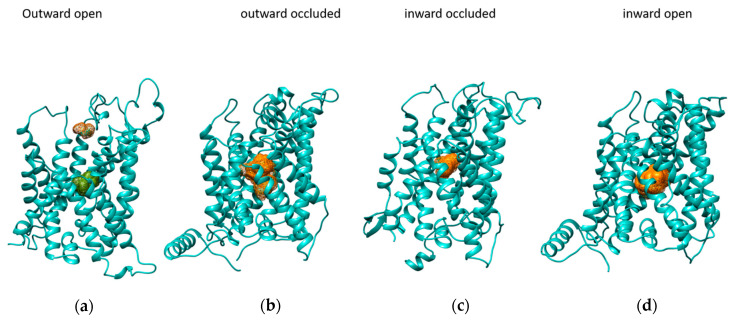
Clusters of histidine in the conformations of LAT1. In the outward open conformation (**a**), there are two clusters, one located in a more external part (orange) and the other (green) in the central part of the protein. There is a single central cluster (orange) in the outward occluded (**b**), inward occluded (**c**), and inward open conformation (**d**), respectively.

To identify the portion of LAT1 involved in the substrate recognition during each step of the transport cycle, blind docking simulations on LAT1 using histidine as a ligand was carried out. Clusters of the substrate have been identified in defined regions of the protein, as shown in Figure 2. These areas are in the central part of the protein in all four conformations, except for the outward-open conformation, which also highlights a cluster located in a more external portion of the transporter. This suggests that a two-step binding event might occur in the outward-open conformation, in which the more external binding site is predicted together with the central cluster (Figure 2a). LAT1 exhibits a transport mechanism similar to that of the bacterial arginine/agmatine antiporter (AdiC). The way histidine interacts with LAT1 E309 at a first glance and then moves in the center of the protein, interacting with several residues among which F252 correlates with the mode of interaction of arginine with AdiC F350, first, and then with W202 [26]. 

**Figure 3:**
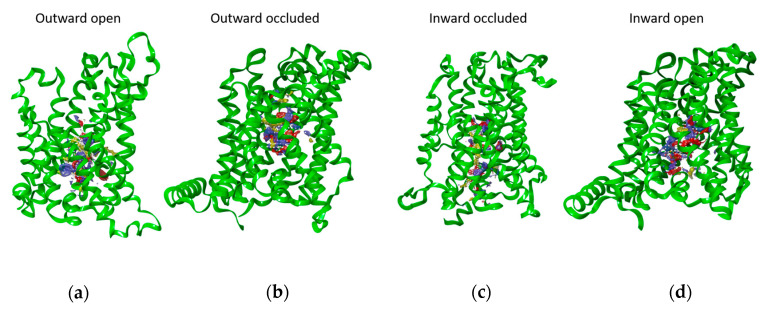
Binding site prediction through SiteMap. The sub-regions of LAT1 in the outward open (**a**), outward-occluded (**b**), inward-occluded (**c**), and inward-open conformation (**d**) that can form bonds or interactions with possible substrates are highlighted using the following color code: Hydrophobic map—yellow mesh, hydrogen-bond donor map—blue mesh, hydrogen-bond acceptor map—red mesh.

**Figure 4:**
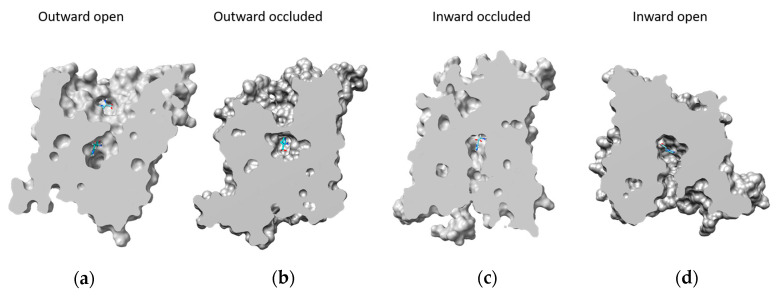
Surface analysis of LAT1 structures. Images show the surface of the transporter in the four different conformations (**a**–**d**). Structure surfaces were “cut” perpendicularly to the lateral view of the structures, by controlling the near clipping planes of the Side View Tool from UCSF Chimera [27]. The extracellular side corresponds to the upper part of the transporter structures.

To confirm that the spatial regions selected by blind docking can be considered reliable, a different computational approach has been adopted. The four structures were explored using SiteMap, an accurate binding site identification tool that can distinguish different sub-regions of protein that can form hydrophobic, polar interaction, or hydrogen bonds with putative substrates. Figure 3 shows the best score putative binding site of LAT1. The identified regions reflect the conformational changes of the transporter. Indeed, except for the more external site, the shape of the SiteMap binding sites is similar to those of the central cavity of LAT1 in the different conformations (Figure 4). The outward-open state is the conformation that facilitates access of the substrate to the binding site. Indeed, in this conformation, the central binding pocket of LAT1 is fully accessible to the solvent (Figure 4a). The subsequent binding of the substrate, such as histidine, causes conformational changes that close the cavity towards the extracellular side (Figure 4b). Further conformational changes must be undergone to move the transporter into a still occluded state, but oriented towards the intracellular side and, thus, called the ‘inward-occluded’ state (Figure 4c). Finally, other conformational changes allow the opening of the cavity towards the intracellular side (Figure 4d). In this inward-open state, the substrate can be released into the intracellular environment. The cycle can then occur in a reverse mode for realizing the export of substrate, according to the antiport mechanism of LAT1. Figure 4 shows the best poses of histidine in each conformational state of the protein obtained by blind docking. Except for the histidine pose in the external binding site of the outward-open conformation, the other histidine molecules bind the transporter mainly in the same subregion.

### 2.2. Docking Analysis

**Figure 5:**
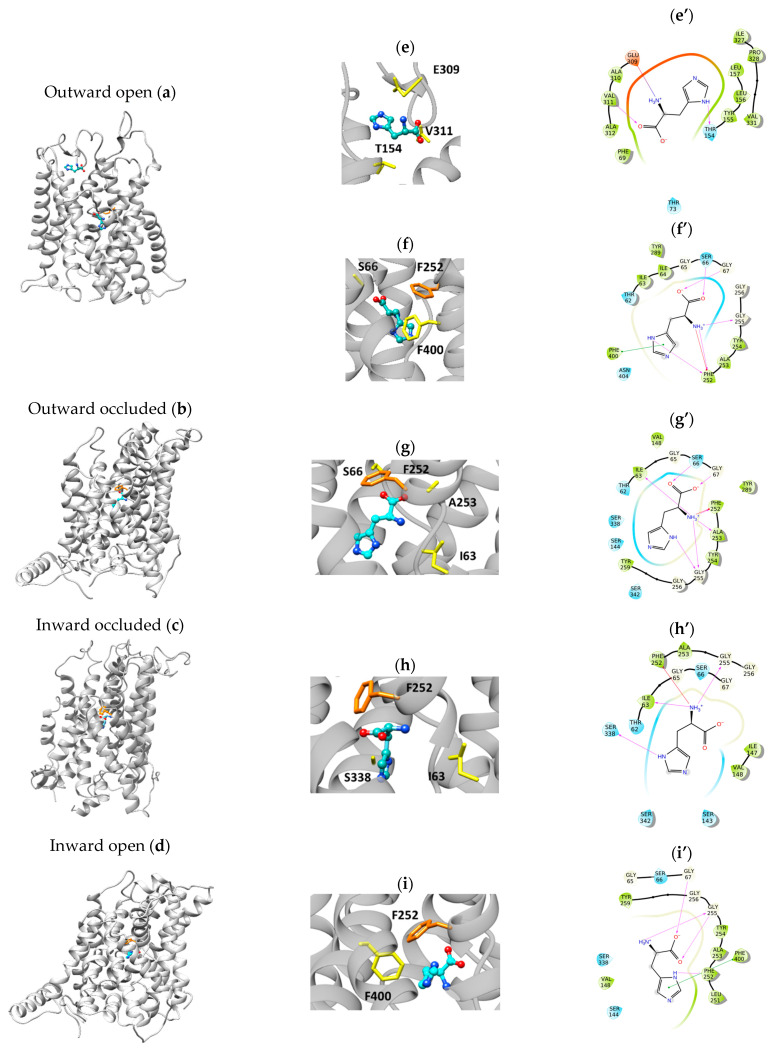
Docking analysis showing the interaction of the substrate with LAT1 in the different conformations. (**a**–**d**), histidine (in cyan), and the F252 (in orange) are shown. (**e**–**i**), zoom of the binding site regions in which residues are involved in the interactions (in yellow), among which F252 (in orange), are shown. (**e’**–**i’**), the 2D view from Maestro of the ligand interaction with the residues corresponding to the 3D (**e**–**i**).

The coordinates from clusters obtained by blind docking were used to generate the grid boxes on each structural conformation of LAT1 to carry out standard precision docking for the 20 amino acids. The histidine path is shown as an example of the substrate/transporter interactions during the transport cycle (Figure 5). The main residue for histidine recognition is F252, depicted in orange in Figure 5. Indeed, this residue was previously identified as crucial for LAT1 activity by site-directed mutagenesis [28]. F252 interacts with or is close to the substrate in all the conformations. In Figure 5, the binding site zoom and the interactions of histidine with the residues predicted by the docking analysis are shown. From the extracellular to the intracellular side, the first step was the outward-open conformation with histidine interacting with the residues E309, V311, and T154 and a Glide Score (G.S.) of −5.72 kcal/mol) (Figure 5a,e,e’). Then, histidine moves down to interact with S66, F252, and F400 (G.S. = −5.09 kcal/mol) (Figure 5a,f,f’). In the second step, which is outward occluded, histidine interacts with I63, S66, G67, F252, A253, and G255 (G.S. = −6.03 kcal/mol) (Figure 5b,g,g’); in the third step, the structure changes to the inward-occluded conformation in which histidine interacts with I63, F252, G255, and S338 (G.S. = −5,31 kcal/mol) (Figure 5c,h,h’); in the fourth and final step that is inward open, histidine interacts with G67, F252, G255, and F400 (Figure 5d,i,i’). In this structure, the histidine position is the closest to the intracellular side (G.S. = −4.93 kcal/mol). Distance values among structure residues and substrate functional groups are reported in the Tables S1 and S2. 

The G.S. data highlight that the best affinity of the transporter for histidine is related to the two occluded conformations. Histidine has been used as the prototype amino acid substrate because it is one of the favorite substrates of LAT1. However, since this transporter also transports other amino acids, we decided to analyze LAT1 interaction with all the 20 amino acids. Table 1 reports the docking G.S. for the 20 amino acids in the four LAT1 conformations obtained by docking analysis for each amino acid. In agreement with the G.S. data for histidine, the two structures, outward occluded and inward occluded, show a higher affinity than the open structures for all 20 amino acids (Table 1). From the G.S. data, it is possible to deduce that LAT1 catalyzes the transport of amino acids with specificity for essential amino acids such as tryptophane, phenylalanine, tyrosine, leucine, isoleucine, valine, and histidine that shows the lower G.S. values. This correlates well with experimental data previously reported [8]. Moreover, the G.S. values of inward-occluded LAT1 for the transported amino acids are less negative than those of the outward-occluded conformation, confirming the functional asymmetry of this transporter previously described based on in vitro experiments [8,17].

### 2.3. Docking of the Inhibitor JPH203

**Figure 6:**
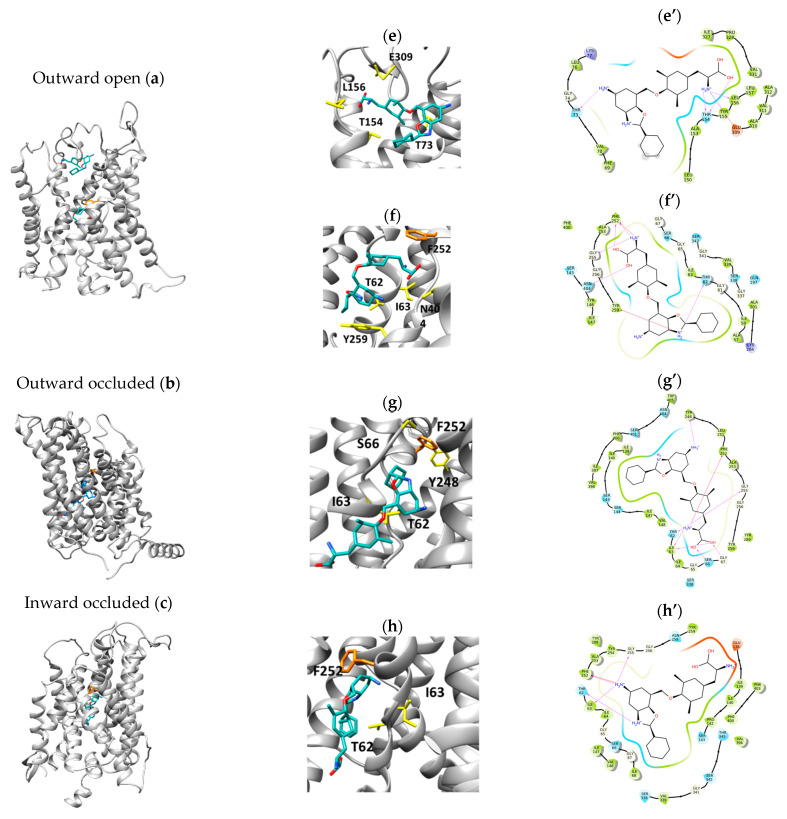

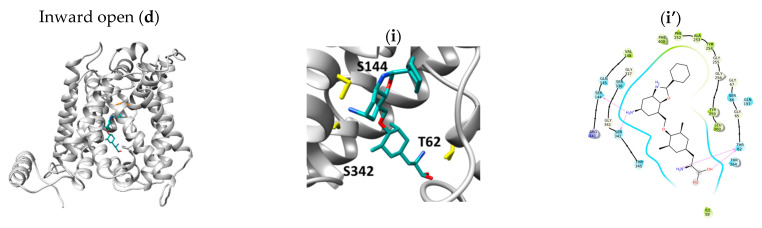
Docking analysis of the inhibitor JPH203. The figure shows the position of JPH203 (in cyan) in the four different conformations (**a**–**d**) and the residue F252 (in orange), the most crucial for the interaction with the inhibitor; the zoom of the binding site regions in the outward-open (**e**,**f**), outward-occluded (**g**) inward-occluded (**h**) and inward-open (**i**) conformations with the residues involved in the interactions (in yellow), among which is F252 (in orange); 2D view from Maestro of the residues predicted to interact with JPH203 in the different conformations (**e’**–**i’**).

A compound with a chemical structure resembling tyrosine, with additional functional groups, was previously identified as a strong inhibitor of LAT1, that is JPH203. This inhibitor is currently under clinical trial as a drug against pancreatic cancer [29]. We performed a docking analysis of the compound in the four LAT1 conformations described in this work. Figure 6 shows the results. The docking simulation performed on the outward open revealed a G.S. of −5.42 kcal/mol for the more external binding site and a G.S. of −7.37 kcal/mol for the central binding site, suggesting that the molecule first meets a lower-affinity place to bind and the second site has a higher affinity (Figure 6a,e,f,e’,f’). The outward-occluded structure shows a G.S. of −8.48 kcal/mol (Figure 6b,g,g’), an inward-occluded G.S. of −7.87 kcal/mol (Figure 6c,h,h’), and an inward open G.S. of −6.49 kcal/mol (Figure 6d,i,i’). [30]. The main residues interacting with the inhibitors are T62, I63, S66, G67, F252, G255, Y259, and S342. Most of these residues correlate well with those of the substrate binding site (see Section 2.2).

## 3. Discussion

**Figure 7:**
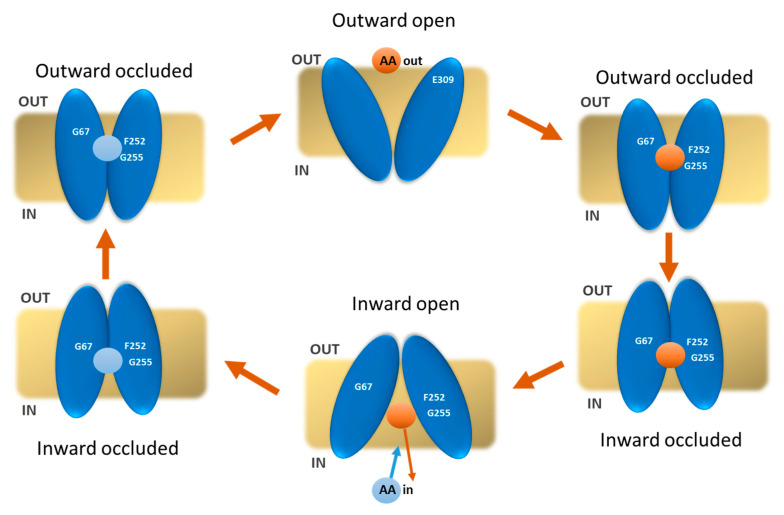
A sketch of the possible His transport cycle of LAT1. In the first step, the transporter is in the outward-open conformation, and the ligand can interact with the highlighted residues; the substrate is bound, and there are conformational changes carrying to the outward-occluded state; the transporter is in the inward-occluded state and in the inward open, ready to release the substrate into the intracellular side. After binding with the substrate, LAT1 returns inward occluded and finally in an outward-occluded state with the release of the substrate in the extracellular side.

The amino acid transporter LAT1 represents a prototype target for cancer therapy since it is over-expressed in many human cancers that derive from tissues in which LAT1 is not physiologically present. Therefore, the identification of inhibitors of LAT1 is an interesting target in drug discovery finalized for cancer therapy. Many research labs worldwide have identified hit compounds by mainly using virtual screening approaches based on docking analysis of large virtual libraries of chemical compounds [31,32,33,34]. In some cases, the identified compounds have also been validated as inhibitors of LAT1 transport activity by using different experimental approaches [35,36,37,38,39,40,41,42,43,44,45,46]. However, the in silico analyses on LAT1 for searching best score compounds have been performed without considering that the transporter undergoes at least four different conformations for accomplishing the transport cycle. The sketch in Figure 7 highlights the main steps of the LAT1 transport cycle. The binding of the substrate on the extracellular side induces conformational changes that, passing through two occluded states with opposite orientations, bring the transporter to open on the intracellular side and release the substrate. After these four steps, an internal substrate molecule binds to the inward open conformation to be transported outside, according to the antiport mode of transport of LAT1. The sketch also highlights that the substrate is locked in the middle of the protein during the transport cycle. Indeed, the alternate substrate exposure on the two “sides” of the membrane is mainly due to the conformational changes of the transporter rather than to the movements of the substrate. Different/variable interactions are expected among the transporter and the compounds depending on the specific conformation of the transporter. To approach this issue, we have analyzed the relationships of the major substrates with the transporter under different conformational states: outward open, outward occluded, inward occluded, and inward open. The various conformations have been directly derived from two available 3D structures of LAT1 or obtained using homology modelling. The relationships of the substrates with the transporter are due to a few residues close to the substrate in all conformations, such as F252. This was revealed as a crucial residue for gating as the residue W202 of AdiC, as revealed by docking and MD [26]. Indeed, the mutation F252A completely suppresses the transport activity, whereas the conservative mutation F252W, resembling the corresponding residue in AdiC, causes only a partial loss of activity [28]. Another bacterial leucine transporter (LeuT) [47,48] that shares the same fold of LAT1, has a phenylalanine residue, namely F253, corresponding to F252 of LAT1. Similarly, the CAT subfamily member *Geobacillus Kaustophilus* amino acid-polyamine-organocation transporter (GkapcT) presents the corresponding F231 as involved in the transport mechanism [49].

Moreover, other interactions are conformation-specific (Figure 5). Some of the residues identified by the proposed approach correspond to previously identified residues [17,22]. 

The affinity of the transporter for the substrates strictly depends on the conformation tested. Interestingly, the in silico analysis highlights that the conformations mainly influencing the affinity are the occluded states, i.e., under conditions of poor, if any, access to the solvent. More negative G.S. values were obtained for these two conformations; between those, the best scores were derived for the outward-occluded conformation. This finding correlates well with previous kinetics analyses in vitro, from which it emerged that the substrate affinity is different on the two “sides” of the membrane [28]. The differences in G.S. under the two different outward-occluded and inward-occluded conformations resemble the differences in Km/Ki observed upon experimental kinetics for the transported substrates previously tested [28]. This is in line with the hypothesis that the substrate/transporter relationships in the two occluded conformations are the major determinants of the substrate affinity. These data highlight that when performing in silico analysis, the conformation issue cannot be neglected. This is very important in the case of drug discovery applications. Indeed, an inhibitor with an apparent low affinity might display a much higher binding capacity if checked in a different transporter conformation and vice versa. As an example of this variability, we have performed the docking analysis of JPH203, which is currently considered the best inhibitor of LAT1, and shows a higher affinity for the occluded conformations, whereas, for the open conformations, the affinity decreases. 

## 4. Materials and Methods

### 4.1. Homology Modelling

To select appropriate templates to build accurate homology models in an outward-open and inward-occluded conformation, the sequence of human LAT1 (Uniprot ID: Q01650) was retrieved from the Uniprot [50] knowledgebase database and used as a query for BLAST against Protein Data Bank (PDB). With the same aim, additional research using the web tools Swiss-Model (Swiss Institute of Bioinformatics Biozentrum, University of Basel, Switzeland) [51], HHpred (Department of Protein Evolution, Max Planck Institute for Biology, Tübingen, Germany) [52], and Phyre2 (Structural Bioinformatics Group, Imperial College, London, UK) [53] was performed. The selected templates for predicting the outward-open state of LAT1 were: 3LRB (3.61 Å) [54], 3NCY (3.2 Å) [55], 5J4I (2.21 Å) [56], 7O82 (1.69 Å) [57], 3OB6 (3 Å) [58], and 3LRC (4 Å) [54]. The selected templates for predicting the inward occluded of LAT1 were: 3GIA (2.32 Å), 3GI8 (2.59 Å), 3GI9 (2.48 Å) [23], 6F34 (3.13 Å), and 5OQT (2.86 Å) [49]. Single or multiple alignments with different template combinations have been generated through PROMALS3D (Howard Hughes Medical Institute, Dallas, TX, USA) [59]. Modeller10.2 (University of California San Francisco, San Francisco, CA, USA) [60] was used to generate a hundred 3D structure models for each alignment. The selection criteria to choose the best model here are briefly described. All the produced models were first evaluated based on the DOPE and GA341 score calculated using Modeller 10.2. Then the best five scored structures were visually inspected and further evaluated using z-DOPE and Ramachandran plots. 

Templates selected to build the outward open homology model structure of LAT1 belonged to the bacterial AdiC transporter and were obtained using crystallography. Structures were taken from RCSB with PDB ID for the apo dimeric structures 3LRB, 3NCY, 5J4I, 7O82, 3LRC, and for the arginine-bound holo dimeric structure 3OB6. Model 58 of 100 from the alignment with the six templates was chosen as the best structure following the above-mentioned criteria.

The arginine-bound 6F34 and alanine-bound 5OQT holo monomeric structures belonging to GkApcT were identified as templates for the inward-occluded homology modeling structure building. After the models were generated, we found that the most-reliable structure was the number 10 (see the above-mentioned criteria). The selected model 10 was subjected to loop refinement using the Modeller Loop Refinement tool.

To produce equilibrated models of LAT1 in the outward-open and inward-occluded conformation, Molecular Dynamics (M.D.) simulations were performed using NAMD (University of Illinois at Urbana-Champaign, IL, USA) [61]. GHARMM-GUI (Lehigh University, Bethlehem, PA, USA) [62] membrane builder input generator was used to insert the selected homology models into a POPC membrane bilayer with 216 lipids for the upper leaf and 213 for the lower leaf. Protein orientation was set up according to the PPM2.0 Server. The system was solvated with water molecules and neutralized with 0.15M of KCl. The system was energy-minimized and then subjected to six equilibration steps before the final production step of 5 ns. Periodic boundary condition and the following parameters were set: 303.15 K and Nose–Hoover thermostat for temperature coupling, 1 bar, and Martyna–Tobias–Klein piston for pressure coupling, and 1 fs as the integration time step. The coordinates and velocities of each atom were saved every 2 ps. The trajectory was analyzed using both VMD (University of Illinois at Urbana-Champaign, IL, USA) [63], and then the trajectory Clustering tool of Chimera was used to determine cluster representatives. The representative structure of the most populated cluster was selected.

### 4.2. Blind Docking

Autodock Vina v1.5.6 (The Scripps Research, La Jolla, California, CA, USA) [64,65] was used to identify histidine-binding sites in the LAT1 transporter using a blind docking procedure. To prepare structures for the subsequent steps of the docking procedure, water molecules, inhibitors, and ligands eventually included in the 3D structures were deleted. Polar hydrogens and Kollman charge were added. Ligands were prepared by adding non-polar hydrogens and charge. After ligand and receptor preparation, a grid box was generated on the whole protein. The box size was 84 × 90 × 110 Å (x, y, and z) with a spacing of 0.5 and an exhaustiveness value of 8. Thirty poses from 3 docking simulations were carried out for each amino acid.

### 4.3. LAT1 Binding Sites Determination and Standard Precision Docking Were Carried Out Using the Schrodinger Maestro Platform

SiteMap: The hydrophobic and hydrophilic regions on the protein surface were investigated using SiteMap from Schrödinger Release 2020-2 (BioLuminate, Schrödinger, LLC, New York, NY, USA) [66], using default parameters and ranked. For each structure, 5 sites were identified using at least 15 site points after generating a fine grid with a grid spacing of 0.35 Å and enabling the detection of shallow binding sites on the surface.

SP Docking: The three-dimensional coordinates of the four conformations of LAT1 were prepared within Maestro 2020-2 using the Schrödinger Protein Preparation Wizard tool, which consists of three essential steps: the addition of hydrogens, the optimization of hydrogen bonds by flipping amino side chains, the correction of charges, and the minimization of the protein complex. Default parameters were used. Ligands were downloaded from PubChem (National Center for Biotechnology Information, Bethesda, MD, USA) [67] in SDF format. Subsequently, they were prepared in Maestro using LigPrep by optimizing the geometries of the ligands and assigning them appropriate protonation states. Epik with default parameters and a pH of 7.0 ± 0.5, which reflects LAT1 environment pH fluctuation, was used. This reflects the physiological environment. Changes in docking at alkaline or acid pH might be expected, which, however, does not have physiological significance. After receptor and ligand preparation, the receptor grids were generated, keeping the default parameters of van der Waals scaling factor to 1.00 and charge cutoff to 0.25 subjected to the OPLS3 force field. A cubic box of specific dimensions (13 Å) centered around the cluster regions revealed by blind docking was generated for the protein. 

Docking analyses were performed using the SP Docking protocol in Maestro. Ten poses were generated for each ligand. 

### 4.4. Visualization of Docking Results

Molecular graphics and visualization of docking results were performed with the UCSF Chimera v.1.7 software (Resource for Biocomputing, Visualization, and Informatics, University of California, San Francisco, CA, USA) [27].

## Figures and Tables

**Table 1:** Docking G.S. (kcal/mol) for the 20 amino acids in the four LAT1 conformations.

Amino Acid	Outward Open	Outward Occluded	Inward Occluded	Inward Open
His	−5.72	−6.3	−5.31	−4.93
Ile	−3.56	−5.15	−4.11	−3.8
Val	−3.77	−5.2	−4.39	−4.14
Leu	−3.59	−5.05	−3.86	−3.79
Phe	−4.57	−5.53	−5.22	−4.85
Cys	−3.85	−4.91	−4.08	−4.01
Met	−3.76	−4.98	−3.79	−3.02
Ala	−2.33	−3.61	−2.56	−2.41
Gly	−1.73	−4.42	−1.94	−1.67
Thr	−3.82	−5.19	−4.78	−3.44
Ser	−3.30	−6.85	−4.16	−3.25
Trp	−4.86	−7.31	−5.86	−5.87
Tyr	−4.07	−5.64	−5.21	−5.49
Pro	−4.35	−5.79	−3.73	−4.75
Glu	−3.84	−5.02	−3.66	−3.48
Gln	−4.09	−5.53	−4.17	−4.08
Asp	−3.99	−5.07	−3.74	−3.77
Asn	−4.44	−5.37	−5.24	−4.24
Lys	−3.79	−4.91	−3.69	−3.74
Arg	−3.05	−4.40	−3.01	−2.63

## Data Availability

The datasets analyzed in this study are available from the corresponding author upon reasonable request.

## Supplementary Materials

The supporting information can be downloaded at: https://www.mdpi.com/article/10.3390/ijms24044042/s1. Reference [68] is cited in supplementary materials.
