# Supplementary material for: Insights into the Transport Cycle of LAT1 and Interaction with the Inhibitor JPH203
Source: Int J Mol Sci. 2023 Feb 17;24(4):4042. doi: 10.3390/ijms24044042 (PMC9965313; doi:10.3390/ijms24044042)
Supplement: Supplementary file 1 [file ijms-24-04042-s001.zip › ijms-2164894-supplementary.pdf]

Table S1 Distances among amino acid residues and functional groups of histidine.

Model conformation	LAT1 amino acid	His functional groups	Distance (Å)
Outward open external site	Thr 154<br>Glu 309<br>Val 311	Ring<br>Amine group<br>Carboxyl group	1.80<br>2.90<br>2.24
Outward open central site	Ser 66<br>Gly 67<br>Phe 252<br>Gly 255<br>Phe 400	Carboxyl group<br>Carboxyl group<br>Amine group<br>Amine group<br>Ring	1.94<br>2.13<br>2.28<br>2.95<br>3.88
Outward occluded	Ile 63<br>Ser 66<br>Gly 67<br>Ala 253<br>Phe 252<br>Phe 252<br>Gly 255<br>Gly 255	Amine group<br>Carboxyl group<br>Carboxyl group<br>Amine group<br>Amine group<br>Amine group<br>Amine group<br>Ring	2.12<br>2.03<br>2.13<br>2.67<br>2.36<br>5.64<br>2.23<br>2.03
Inward occluded	Ile 63<br>Phe 252<br>Gly 255<br>Ser 338	Amine group<br>Amine group<br>Amine group<br>Ring	2.17<br>3.15<br>4.20<br>2.80
Inward open	Gly 67<br>Phe 252<br>Gly 255<br>Gly 255<br>Phe 400	Carboxyl group<br>Ring<br>Amine group<br>Carboxyl group<br>Ring	2.41<br>1.97<br>2.61<br>2.28<br>4.44

Table S2 Distances among amino acid residues and JPH203 functional groups identified as in the article

Model conformation	LAT1 amino acid	JPH203 functional group	Distance (Å)
Outward open external site	Thr 73	Benzo oxazole	2.87
	Thr 154	Carboxyl group	2.06
	Thr 154	Amine group	2.75
	Leu 156	Amine group	1.74
	Glu 309	Amine group	4.74
Outward open central site	Thr 62	Benzo oxazole	2.06
	Phe 252	Amine group	1.57
	Phe 252	Amine group	1.72
	Phe 252	Carboxyl group	2.13
	Gly 255	Amine group	3.06
	Tyr 259	Benzo oxazole	4.53
	Asn 404	Carboxyl group	3.21
Outward occluded	Thr 62	Amine group	2.06
	Ile 63	Amine group	2.19
	Ile 63	Carboxyl group	1.85
	Ser 66	Carboxyl group	2.41
	Gly 67	Carboxyl group	2.66
	Tyr 248	Benzo oxazole	2.18
	Phe 252	Amine group	3.00
	Gly 255	Amine group	2.47
Inward occluded	Thr 62	Benzo oxazole	4.56
	Ile 63	Benzo oxazole	2.05
	Phe 252	Benzo oxazole	2.69
	Phe 252	Benzo oxazole	5.39
	Gly 255	Benzo oxazole	2.83
Inward open	Thr 62	Carboxyl group	1.94
	Thr 62	Amine group	2.13
	Ser 144	Benzo oxazole	2.43
	Ser 342	Benzo oxazole	1.62

1. Achmad, A.; Lestari, S.; Holik, H.A.; Rahayu, D.; Bashari, M.H.; Faried, A.; Kartamihardja, A.H.S. Highly Specific L-Type Amino Acid Transporter 1 Inhibition by JPH203 as a Potential Pan-Cancer Treatment. *Processes* **2021**, *9*, 1170. https://doi.org/10.3390/pr9071170
